# Impact of occlusal stabilization splints on global body posture: a prospective clinical trial

**DOI:** 10.1007/s00784-024-05888-9

**Published:** 2024-08-29

**Authors:** Tristan Hampe, Laura Fürstberger, Tobias L. Kordsmeyer, Lars Penke, Alannah M. Mahler, Clemens M. Mäder, Ralf Bürgers, Sebastian Krohn

**Affiliations:** 1 Department of Prosthodontics, University Medical Center Göttingen, 37075 Göttingen, Germany; 2 Department of Psychology & Leibniz ScienceCampus Primate Cognition, University of Göttingen, 37073 Göttingen, Germany; 3 Department of Orthodontics, University Medical Hospital Regensburg, 93053 Regensburg, Germany

**Keywords:** Body posture, Temporomandibular disorders, Craniocervical posture, 3D scanning, Stabilization splint

## Abstract

**Objectives:**

Body posture of patients with temporomandibular disorders (TMD) has been investigated using different methods, whereas outcome and conclusions were controversial. The present clinical trial aimed to investigate the effects of splint therapy on global body posture.

**Materials and methods:**

24 subjects (20 females, 4 males; age 24.2 ± 4.0 years) with TMD symptoms were examined clinically (RDC/TMD) and subsequently, splint fabrication was initiated. Along with routine therapy, all subjects underwent three-dimensional pre- and post-treatment full body scans in standing and upright sitting posture using a Vitus Smart XXL 3D scanner. Each scan was acquired in triplicate and evaluated in duplicate, measuring twelve standing and nine sitting postural parameters. Influencing factors were analyzed using analysis of variance (ANOVA), and additional Bland-Altman analyses verified the significance of the ANOVA results.

**Results:**

The increase of Forward Head angles and the decrease of Round Shoulders angles were consistent for both positions and sides. Forward Head angles were significantly influenced by limited mandibular mobility and myofascial pain. Round Shoulders angles showed a significant correlation with myofascial pain, joint noises and the absence of limited mandibular mobility.

**Conclusion:**

The influence of occlusal splints on global posture is limited and only small effects on cervicocranial parameters were found. In the present study, the average head position of post treatment measurements was more centered on the body’s core, whereas the shoulders were tilted more anteriorly.

**Clinical relevance:**

Understanding the limited influence of occlusal splints on cervicocranial parameters underscores the need for multimodal treatment strategies for TMD patients.

## Introduction

In healthy humans, the equilibrium of musculoskeletal alignment allows them to resist gravitational forces during standing and upright sitting with minimal muscle activity [1]. Musculoskeletal alterations of the head and neck region are summarized by the umbrella term of Temporomandibular disorders (TMD) [2]. Cardinal symptoms of TMD are restrictions of the vertical or horizontal mandibular range of motion, sounds of the Temporomandibular joint (TMJ), and pain of the masticatory muscles or TMJ [3]. This disorder of the masticatory system is classified into a wide range of diagnostic subgroups that include more common TMDs like masticatory muscle disorders, joint pain or disc disorders and less common TMDs such as congenital disorders [4]. TMDs may negatively affect sleep quality and life satisfaction and epidemiological studies suggest that TMD is predominant in females with an age of 25 to 44 years [5] whereas detectable degenerative radiographic findings of the TMJ seem to be associated with increased age and male gender [6].

Due to anatomical proximity, biomechanical relationships as well as neurophysiological connections, recent literature suggests associations between TMD, back pain [7, 8], or tension-type headaches [9]. This has led to a growing interest in exploring the link between orthopedic alterations of body posture and TMD symptoms [10]. The comprehensive treatment of TMD includes the use of occlusal splints to reduce subjective pain by reorganizing neuromuscular-functional patterns and inhibiting regional overloading of chewing muscles [11–14]. To test the hypothesis that TMD therapy influences body posture, several studies have investigated the outcomes of TMD therapy on distant body segments [15–17]. However, due to the low reliability and inaccuracy of the devices used to measure postural changes [18], reliable prospective studies investigating the effect of TMD treatment on other body segments are still lacking [19]. Therefore, recent systematic reviews conclude that there is insufficient scientific evidence, to support the thesis of interactions of TMD treatment with postural changes [19–21].

But recent developments in 3D-body scanning enable the quick non-invasive acquisition of full-body surface data in a very accurate manner [22, 23]. A complementary applicable evaluation method for three-dimensional body scan data proposed by Tomkinson and Shaw is suggested to show good repeatability and low technical error in determining postural changes [24, 25]. This method allows for multiple scans of a single subject within one day, reducing the impact of diurnal posture variability, and enabling the collection of reliable posture data [25].

Utilizing this novel body posture evaluation method of 3D body scans, this prospective study aimed to investigate the influence of splint treatment on global body posture of TMD patients. It is the first study to examine the effects of occlusal splints on specific postural angles in TMD patients using a non-invasive and rapid detection technique to evaluate global body posture.

## Materials and methods

### Participants

This prospective, clinical trial was approved by the Institutional Ethics Committee of the University Medical Center Göttingen (application number 22/7/15) and executed in accordance with the principles of the Declaration of Helsinki. All patients participated in the trial on a voluntary basis after receiving comprehensive information about the aim and design of the study and signing an informed consent form. This report complies with the STROBE guidelines for observational studies.

The patients were recruited at the department of prosthodontics of the University Medical Center Göttingen during TMD consultations. Inclusion and exclusion criteria are listed in Table 1. 24 TMD patients with a mean age of 24.3 (± 3.9 years) were examined clinically according to RDC/TMD criteria [26] by a specialist of the German Society for Functional Diagnostics and Therapy (DGFDT) with more than ten years of experience. The study group consisted of 20 female and 4 male subjects. Further statistical processing was ensured by simplification of the results of clinical examination by pooling diagnoses into groups, as already proposed by other authors [27]. Subjects, which solely showed pain of chewing muscles, while TMJ examination showed normal results, were summarized in the Myofascial Pain Group (RDC/TMD group I: Myofascial pain with / without limited opening). The Combined Pain Group consisted of participants with both myofascial pain and painful alterations of the TMJ (RDC/TMD group I plus a diagnosis from group II: Disc Displacement with / without reduction or group III: arthralgia, osteoarthritis). Routine splint treatment was applied to all subjects, whereas additional investigations of the present study design did not interfere with TMD therapy. In order to initiate occlusal splint processing conventional impressions were taken using alginate (Palgat Plus, 3 M Espe, Seefeld, Germany). The bite was registered using a silicone-based bite registration material (Registrado X-tra, VOCO, Cuxhaven, Germany) and the arbitrary hinge axis was determined with a facebow (Axioquick, SAM, Gauting, Germany). Subsequently, stone casts were manufactured, and on the upper casts, a paraocclusal tray was made from light-polymerized acrylic resin following the manufacturer’s recommendations. On the next appointment, centric relation was recorded, using the acrylic base with a front jig and the same bite registration material (Registrado X-tra, VOCO, Cuxhaven, Germany). Prior to bite registration, neuromuscular deprogramming was performed using an Aqualizer ultra medium (Aqualizer Splint Systems, Bainbridge Island, USA) for ten minutes. This procedure resulted in a free intermaxillary space of approximately 2–5 mm (depending on the region within the dental arch). Within this space, maxillary Michigan splints were fabricated using a thermoforming plate (Erkodur 1 mm; Erkodent, Pfalzgrafenweiler, Germany) that covered all teeth and served as a splint base. Cold-cured polymethyl methacrylate (PMMC) (Weitur; Weithas, Lütjenburg, Germany) was applied for the adjustment of the equilibrated occlusion in the articulator. The prepared splints were polished, and additionally, the occlusal surfaces were adjusted intraorally to ensure equilibration of static occlusion and anterior/canine guidance. Prior to the application of the occlusal appliances, all subjects were fully instructed regarding splint handling, application time (during the night) and duration of intervention. Treatment adherence was estimated on the basis of self-reported duration of splint application, since adherence could not be evaluated as proposed in prior studies [28–30]. After a treatment duration of three months, potential postural changes were determined by post treatment 3D body scans.

**Table 1:** Inclusion and exclusion criteria

Inclusion criteria	Exclusion criteria
TMD diagnosis according to RDC/TMD requiring splint treatment	No TMD diagnosis
Age > 18	TMD therapy doesn’t require splint treatment
No pre-existing orthopedic condition	Patient was already treated with a splint or is treated outside the department of prosthodontics of the University Medical Center Göttingen
Signed written informed consent form	Age < 18
Body height < 2,1 m	No consent to participate in the study
	Pre-existing orthopedic conditions
	Body height > 2,1 m

### Analysis of global body posture

**Fig. 1:**
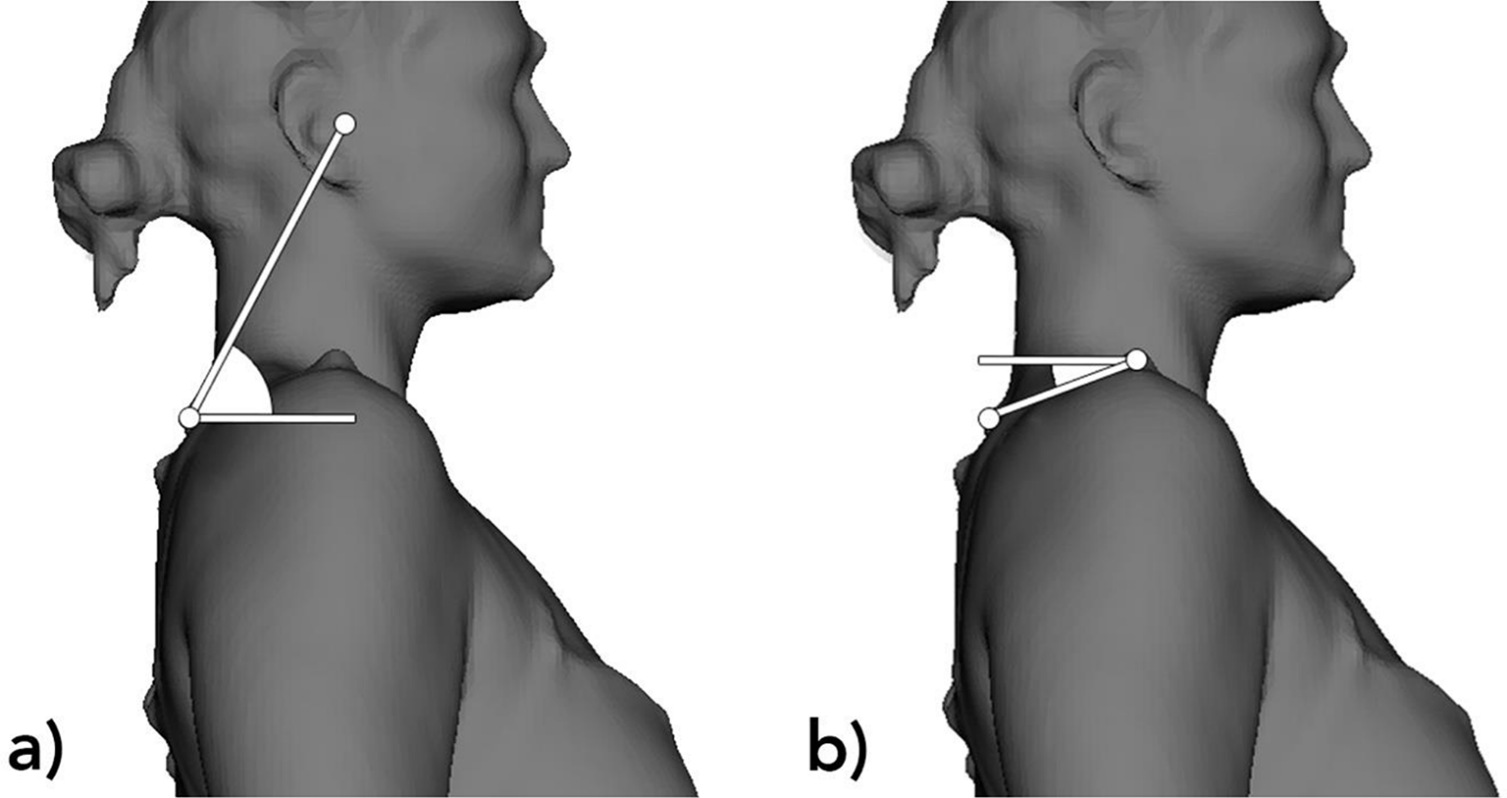
3D-Scan acquired with the Vitus Smart XXL 3D scanner. (**a**) Forward Head: The angle is formed by the intersection of a line joining the tragus and the spinous process of C7, and a projected line on a Transverse plane at the level of C7. (**b**) Round Shoulders: The angle is formed by the intersection of a line joining C7 and the acromion, and a projected line on a Transverse plane at the level of the acromion

Three-dimensional surface scans were generated before and after splint therapy (timepoints: T1/T2) by a Vitus Smart XXL laser scanner (Human Solutions, Kaiserslautern, Germany) (see the specification listed in Table 2). The scans were performed by two physical therapists with more than five years of experience in the field at the Department of Psychology & Leibniz ScienceCampus Primate Cognition of the University of Göttingen. In order to reveal potential functional changes in body posture due to splint treatment, all scans were carried out in both standing and upright sitting positions to rule out primary skeletal confounding factors, such as bony discrepancies in leg length. Reliability and validity of measurements were guaranteed by using scan markers (Human Solutions, Kaiserslautern, Germany). For the ideal positioning of the physical and digital markers, all subjects wore form-fitting underwear during the scans. The markers were attached to prominent bony reference points prior to all full body scans by two trained observers, who were blinded to TMD diagnoses. Subjects were asked to assume their “normal” standing position for landmarking and scanning, which was found by: taking a few steps in place to find their normal foot position, moving their head and neck to look down, then up, then straight ahead to find their normal head position. To find their “normal” sitting position subjects were asked to perform the same sequence of motions with their head as they did for the standing position. Motion artifacts were successfully ruled out by asking the participants to stand as still as possible during the scans, which took about 10 s. Reproducibility of the aforementioned method was evaluated by conduct of repeated measurements, i.e. each scan was carried out in triplicate (S1, S2, S3). All scans were recorded without intraoral splint application to avoid confounding effects on body posture due to occlusal changes. The evaluation of specific postural parameters was performed in frontal as well as sagittal orientation and included a variety of different angles as described by Tomkinson and Shaw (see Tables 3 and 4; Fig. 1) [24] by using Anthroscan Software (Human Solutions, Kaiserslautern, Germany). The same evaluation method was adapted and used for the measurement in the sitting position. Three dimensional coordinates (Cartesian (XYZ) coordinates) of each landmark were transferred by using extensible markup language file format (XML) to standardized Excel spreadsheets (Microsoft, Redmond, Washington, USA) to calculate potential alterations in posture-related angles.

**Table 2:** Technical specifications of the VITUS smart XXL

Measurement technique	optical triangulation with laser light
**Sensor heads**	4 dual-camera heads
**Safety**	Eye-safe Laser Class 1
**Measurement range Height**	2,1 m
**Measurement range Depth**	1,0 m
**Measurement range Width**	1,2 m
**Approx. measurement time**	10 s
**Scanner Area**	4.84 m^2^

**Table 3:** The description of the evaluated landmarks as described by Tomkinson and Shaw [24]

Landmark	Description
Infraorbital rim	Most caudal point of the orbital contour
Tragus	Central point of tragus
Acromiale	Highest and most lateral point on the acromion process of the scapula
ASIS	Most prominent point on the anterior superior iliac spine (ASIS)
C7	Most prominent posterior point on the spinous process of the seventh cervical vertebra (C7)
T3	Most prominent posterior point on the spinous process of the third thoracic vertebra (T3)
T7	Most prominent posterior point on the spinous process of the seventh thoracic vertebra (T7)
T12	Most prominent posterior point on the spinous process of the twelfth thoracic vertebra (T12)
L3	Most prominent posterior point on the spinous process of the third lumbar vertebra (L3)
PSIS	Most prominent point on the posterior superior iliac spine (PSIS)
S2	Most prominent posterior point on the spinous process of the second sacral vertebra (S2)
Greater trochanter	Most lateral point on the greater trochanter of the femur
Lateral Femoral Epicondyle	Most lateral point on the lateral epicondyle of the femur
Mid-patella	Central point on the anterior surface of the patella
Tibial tuberosity	Central point of the tibial tuberosity
Lateral Malleolus	Most lateral point on the distal protrusion of the fibula at the ankle

**Table 4:** The definition, description, and interpretation of each of the postural measurements as described by Tomkinson and Shaw [24]

Postural measurement	Definition	Description	Landmarks	Interpretation
*Sagittal plane*				
Frankfort plane	The degree to which the head is tilted forward or backward	The included angle formed by the intersection of a line connecting the lowest point on the inferior margin of the orbit to the tragus of the ear, and a projected line on a transverse plane at the level of the tragus, extending from the tragus to a perpendicular line from the plane to the lowest point on the inferior margin of the orbit.	Infraorbital rim (R), Tragus (R)	A positive angle indicates the head is tilted forward, while an angle of 0° corresponds to a straight head position. Negative angles indicate a posterior tilt.
Forward head*	Degree to which the head is inclined forward	The included angle formed by the intersection of a line connecting the tragus of the ear to the spinous process of C7, and a projected line on a transverse plane at the level of C7, extending from C7 to a perpendicular line from the plane to the tragus.	Tragus (R), C7	A smaller angle indicates a more pronounced forward head posture.
Round shoulders*	Degree to which the shoulders are inclined anteriorly	The included angle formed by the intersection of a line connecting the spinous process of C7 to the acromion, and a projected line on a transverse plane at the level of the acromion, extending from the acromion to a perpendicular line from the plane to C7.	C7, Acromiale (R), Acromiale (L)	A smaller angle indicates a more pronounced forward shoulder posture.
Thoracic kyphosis*	Degree of posterior curvature of the thoracic spine	The included angle formed by the intersection of a line connecting the spinous process of C7 to the peak of the thoracic spinal curve, and a line connecting the peak to the spinous process of T12.	C7, T3, T7, T12	A smaller angle indicates a greater posterior curvature of the thoracic spine.
Lumbar lordosis*	Degree of anterior curvature of the lumbar spine	The included angle formed by the intersection of a line connecting the spinous process of T12 to the peak of the lumbar spinal curve, and a line connecting the peak to the spinous process of S2.	T12, L3, S2	A smaller angle indicates a greater anterior curvature of the lumbar spine
Pelvic tilt	The degree of anterior or posterior tilt of the pelvis.	The included angle formed by the intersection of a line connecting the PSIS and ASIS, and a projected line on a transverse plane at the level of ASIS, extending from ASIS to a perpendicular line from the plane to PSIS.	ASIS (R), PSIS (R)	A positive angle indicates an anterior pelvic tilt, while a smaller angle indicates a less pronounced tilt and negative values indicate a posterior tilt.
Knee flexion/extension*	The degree of knee flexion or extension	The included angle formed by the intersection of a line connecting the greater trochanter to the lateral epicondyle of the femur, and a line connecting the lateral epicondyle to the lateral malleolus, when viewed laterally.	Greater trochanter (R), Greater trochanter (L), Femoral epicondyle lateral (R), Femoral epicondyle lateral (L), Malleolus lateral (R), Malleolus lateral (L)	An angle greater than 180° indicates knee flexion, with an angle close to 180° indicates less to no knee bending. Angles below 180° indicate a knee extension.
*Coronal plane*				
Head alignment	The degree of lateral head tilt.	The included angle formed by the intersection of a line connecting the lowest points on the inferior margins of the left and right orbits, and a projected line on a transverse plane at the level of the lowest point on the inferior margin of the right orbit connected to a perpendicular line from the inferior margin of the left orbit.	Infraorbital rim (R), Infraorbital rim (L)	A positive angle indicates that the head is tilted down to the left, an angle of 0° corresponds to no tilt, and a negative angle indicates that the head is tilted down to the right.
Shoulder alignment	The degree of bilateral shoulder tilt.	The included angle formed by the intersection of a line connecting the left and right acromia, and a projected line on a transverse plane at the level of the right acromion connected to a perpendicular line from the left acromion.	Acromiale (R), Acromiale (L)	A positive angle indicates that the shoulders are tilted down to the left, an angle of 0° corresponds to no tilt, and a negative angle indicates that the head is tilted down to the right.
Pelvis alignment	The degree of lateral pelvic tilt.	The included angle formed by the intersection of a line connecting the left and right ASIS, and a projected line on a transverse plane at the level of the right ASIS connected to a perpendicular line from the left ASIS.	ASIS (R), ASIS (L)	A positive angle indicates that the pelvis is tilted to the left, an angle of 0° corresponds to no tilt, and a negative angle indicates that the head is tilted down to the right.
Quadriceps angle*	The degree of the quadriceps’ superior and lateral pull.	The angle formed by the intersection of a line connecting ASIS to the midpoint of the patella, and the extension of a line connecting the midpoint of the patella to the midpoint of the tibial tuberosity.	ASIS (R), ASIS (L), Mid-patella (R), Mid-patella (L), Tibial tuberosity (R), Tibial tuberosity (L)	A smaller angle indicates a more superior pull of the quadriceps.
Genu valgum/varum*	The degree of lateral (valgum) or medial (varum) angulation of the tibia relative to the femur.	The angle formed by the intersection of a line connecting the greater trochanter to the lateral epicondyle of the femur, and a line connecting the lateral epicondyle to the lateral malleolus, when viewed anteriorly.	Greater trochanter (R), Greater trochanter (L), Femoral epicondyle lateral (R), Femoral epicondyle lateral (L), Malleolus lateral (R), Malleolus lateral (L)	An angle greater than 180° indicates medial (varum) angulation, while an angle closer to 180° indicates less deviation of the tibia.

R = right; L = left; ASIS = Anterior superior iliac spine; PSIS = Posterior superior iliac spine; C7 = 7th cervical vertebra; T3 = 3rd thoracic vertebra; T7 = 7th thoracic vertebra; T12 = 12th thoracic vertebra; L3 = 3rd lumbar vertebra; S2 = 2nd sacral vertebra. *Postural measurements calculated in absolute angular degrees (°)

Postural measurements in the Sagittal plane (Table 4) require only the X and Z coordinates of the corresponding landmarks, and those in the Coronal plane require only the Y and Z coordinates. The calculation of three different kind of angles was performed: (A) Calculation of an angle between a line joining two landmarks and a projected plane at the level of one landmark; (B) Calculation of an angle between a line joining two landmarks and a second line from one landmark to a third landmark; (C) Calculation of an angle between a line joining a measured and a derived landmark, and a line from the derived landmark to another measured landmark.

**Fig. 2:**
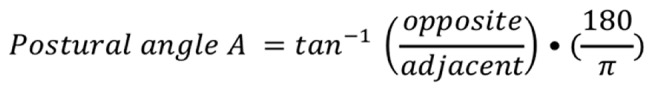
Mathematical formula for the calculation of an included angle between a line joining two landmarks and a projected plane at the level of one landmark

The calculation of an included angle between a line joining two landmarks and a projected plane at the level of one landmark (A) requires the X and Z (sagittal) or the Y and Z coordinates (coronal) of two measured landmarks and one derived landmark on a transverse plane. The landmarks form a right-angled triangle, with the opposite side being the Z coordinate difference and the adjacent side being the X coordinate difference. Trigonometry is then used to determine the angle for the postural measurement (see Fig. 2).

**Fig. 3:**
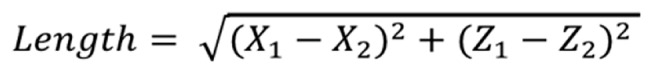
Mathematical formula for the calculation of the side lengths (**a**, **b** and **c**) of the respective triangle. For the calculation of coronal angles, the X coordinates are replaced by Y coordinates

**Fig. 4:**
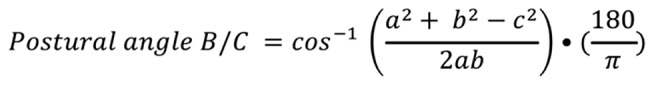
Mathematical formula for the calculation of the postural angle using the calculated side lengths of the triangle

The calculation of an included angle between a line joining two measured landmarks and a second line connecting one of these landmarks with a third landmark (B) requires the coordinates of three measured landmarks. For the calculation of coronal parameters, the X coordinates are replaced by Y coordinates. These three landmarks form a triangle, with sides a, b, and c representing the distances between each pair of landmarks. The lengths of these sides are calculated using their respective coordinates (Fig. 3). The resulting side lengths are then used to calculate the angle describing the postural measurement (Fig. 4). For the quadriceps angle the resultant angle is subtracted from 180°.

The calculation of an included angle formed between a line joining one measured and one derived landmark, and a second line joining the derived landmark to another measured landmark, requires the coordinates of two measured landmarks and one derived landmark (C). This applies to the calculation of the sagittal parameter’s thoracic kyphosis and lumbar lordosis. Using the X and Z coordinates of these landmarks to construct a triangle, Cartesian coordinate geometry and trigonometry are used to calculate the postural measurement. The coordinates of the peak of the spinal curve are determined using the D_max_ procedure as described by Cheng et al. [31], which fits a polynomial curve to the spinal coordinates (swapping X and Z). The derived landmark forms a triangle with the two end landmarks, and the side lengths are calculated using their coordinates (Fig. 3). These lengths are then used to determine the postural measurement angle (Fig. 4).

Analyses of all scans were performed twice (M1/M2) by one trained observer, who was blinded to both the clinical diagnoses and the timepoints (T1/T2).

### Statistical analyses

Statistical evaluation of the acquired data included graphical processing, which was performed using XLSTAT (Addinsoft, Paris, France). Before conducting statistical analyses, requirements for the use of parametric tests were enrolled by descriptive statistics and Shapiro-Wilk tests. Subsequently, test-retest reliability for duplicate measurements (M1/M2) was determined by using Spearman’s rank correlation coefficient in accordance with the following allocation: *R* = 0–0.29 / 0.30–0.49 / 0.50–0.69 / 0.70–0.99 / 1.00. These values corresponded to no / weak / moderate / strong or perfect linear correlation. Since presence and amount of changes between T1 and T2 were of great importance, each angle suggested by Tomkinson and Shaw, was investigated separately for both sitting and standing position by utilization of Bland-Altman analyses. Consequently, new datasets were created, which solely included sequences showing postural changes that were consistent for both sides and both positions. Further statistical analyses regarding influencing factors, such as gender, TMD diagnoses, joint noise, or mandibular mobility, were performed by applying analysis of variance (ANOVA). In case of significance, additional Bland-Altman analyses were applied in order to verify the validity of significant differences.

## Results

Clinical examinations revealed 15 subjects with combined pain and 9 participants with myofascial pain. Treatment of all participants proceeded uneventfully, and participants mainly stated that they used their splints overnight for 7 to 8 h.

**Fig. 5:**
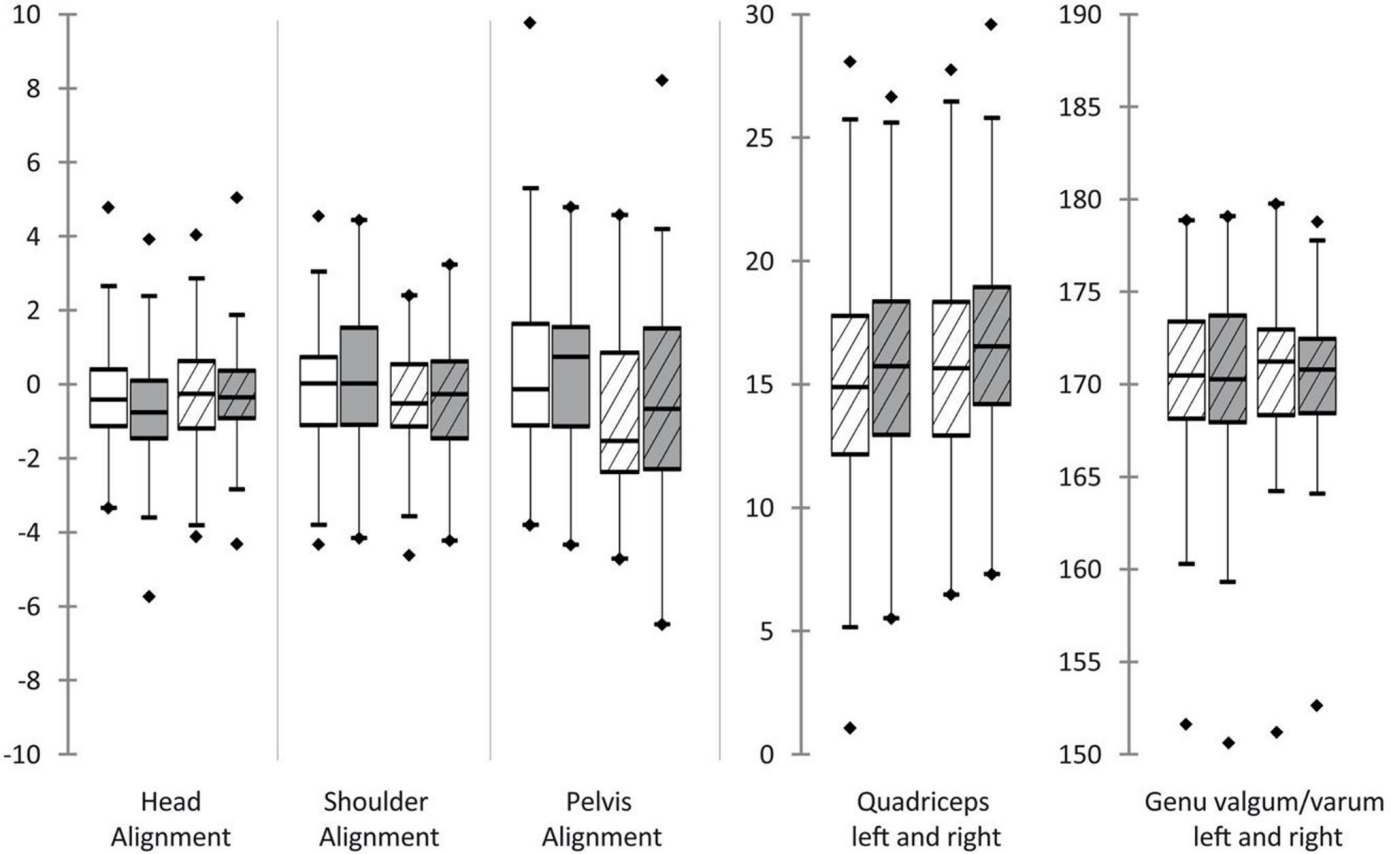
All measured angles in the coronal plane before and after splint therapy. White: before splint therapy (T1); Grey: after splint therapy (T2); Without stripes: sitting; Striped: standing; *Highlighted changes were significant and coherent for both sides and positions

**Fig. 6:**
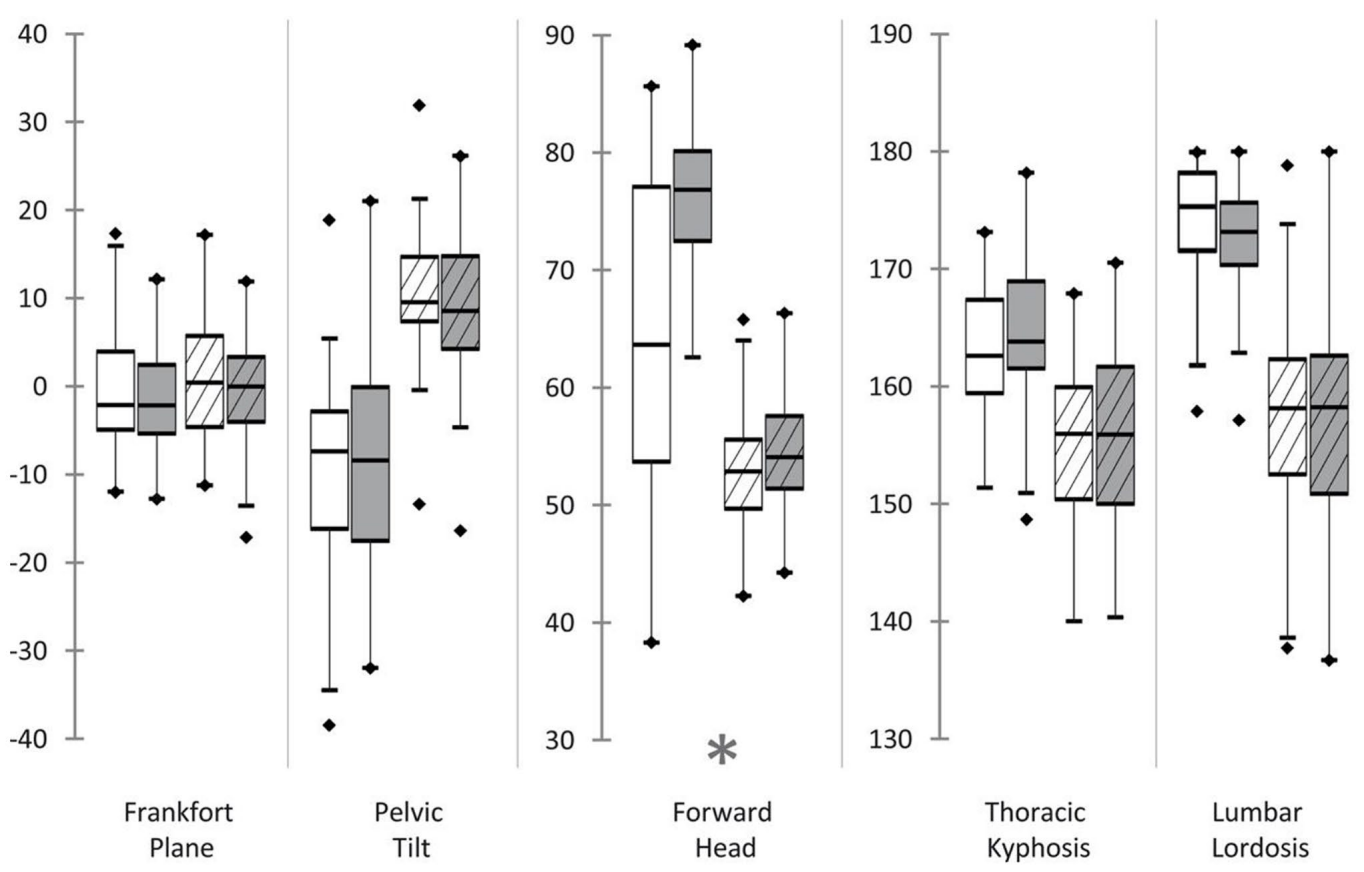
Measured angles of the body core in the sagittal plane before and after splint therapy. White: before splint therapy (T1); Grey: after splint therapy (T2); Without stripes: sitting; Striped: standing; *Highlighted changes were significant and coherent for both sides and positions

**Fig. 7:**
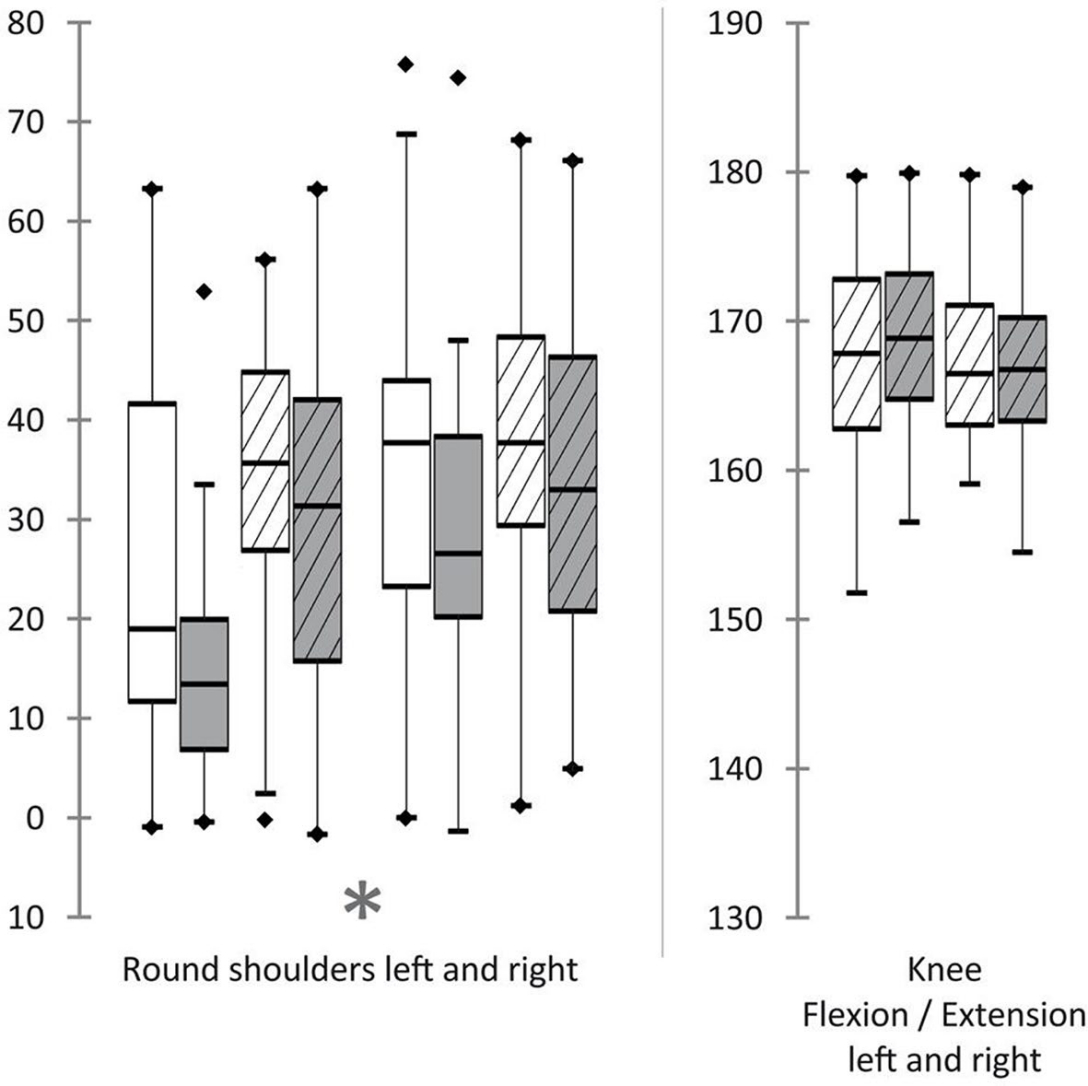
Measured angles of the extremities in the sagittal plane before and after splint therapy. White: before splint therapy (T1); Grey: after splint therapy (T2); Without stripes: sitting; Striped: standing; *Highlighted changes were significant and coherent for both sides and positions

Acquisitions of three-dimensional full body scans were successful in all participants. In total 288 scans were recorded, whereas each subject was scanned in triplicate (M1/M2/M3) at two points in time (T1/T2) in two body positions (sitting/standing). Analyses of each scan were enrolled twice in order to calculate test-retest reliability. The results of the Shapiro-Wilk tests indicated that none of the resulting angles followed a normal distribution. Test-retest reliability for all duplicate measurements was strong (*R* = 0.7–0.99; see Table 5). In standing position, Bland-Altman analyses revealed relevant differences between angles at T1 and T2 for Forward head, Round shoulders left and right, Pelvis alignment and Quadriceps angle right. The scans of sitting positions showed relevant differences between T1 and T2 for Forward head, Round shoulders left and right, Thoracic kyphosis, Lumbar lordosis, Pelvic tilt and Head alignment. Table 6 lists all median angles and differences between T1 and T2. Only the increase of the Forward head angles and the decrease of the Round shoulders (left/right) angles were consistent for both positions and sides (see Figs. 5, 6 and 7). Therefore, further statistical analyses regarding influential factors were performed on the basis of new datasets, which solely included the series of consistent results of both positions. For this purpose, data of both positions, and if applicable of both sides was pooled and filtered for participants with a decrease in Round shoulders or an increase in Forward head, which was interpreted as being influenced by application of occlusal splints. Analysis of variance (ANOVA) was applied to evaluate the influence of gender, the presence or absence of limited mandibular mobility and joint noises. Furthermore, the difference between myofascial pain and combined pain was investigated. The ANOVA results revealed significant negative influence of the presence of myofascial pain and limited mandibular mobility on the values of Forward head angles (*p* < 0.0001; *p* < 0.0001). The Forward head angles were also influenced by the standing position (*p* < 0.0001). Round shoulders angles showed a decrease from T1 to T2, which were significantly influenced by the diagnosis of myofascial pain (*p* < 0.0001) as well as the absence of limited mandibular mobility (*p* < 0.0001) and the presence of joint noises (*p* = 0.043) to more negative values.

**Table 5:** Calculation of test-retest-reliability by utilization of Spearman’s rank correlation coefficient

Value	Spearmann’s correlation coefficient
Sagittal Parameters, standing	0.993
Coronal Parameters, standing	0.982
Sagittal Parameters, sitting	0.990
Coronal Parameters, sitting	0.799

**Table 6:** Average changes of posture after splint treatment and results of bland-Altman analyses (BA). Parameters showing relevant changes in both sitting and standing position are marked (*)

Parameter			Median angle before therapy (in degrees)	Median angle after therapy (in degrees)	Difference between the timepoints (in degrees)	Relevant change according to BA
**Sagittal** **Plane**	Frankfort Plane	Standing	0.42	0.00	-0.42	**yes**
		Sitting	-2.14	-2.21	-0.07	no
	*Forward head	Standing	52.99	54.18	+ 1.19	**yes**
		Sitting	63.66	76.85	+ 13.19	**yes**
	*Round shoulders right	Standing	37.76	33.02	-4.74	**yes**
		Sitting	37.71	26.57	-11.14	**yes**
	*Round shoulders left	Standing	35.84	31.54	-4.30	**yes**
		Sitting	19.19	13.63	-5.56	**yes**
	Thoracic kyphosis	Standing	155.94	155.89	-0.05	no
		Sitting	162.60	163.82	+ 1.22	**yes**
	Lumbar lordosis	Standing	158.13	158.22	+ 0.09	no
		Sitting	175.31	173.16	-2.15	**yes**
	Pelvic tilt	Standing	9.51	8.50	-1.01	no
		Sitting	-7.38	-8.42	-1.04	**yes**
	Knee flexion / extension right		166.46	166.75	+ 0.29	no
	Knee flexion / extension left		167.84	168.86	+ 1.02	no
**Coronal** **Plane**	Head alignment	Standing	-0.25	-0.34	-0.09	no
		Sitting	-0.41	-0.75	-0.34	**yes**
	Shoulder alignment	Standing	-0.53	-0.28	+ 0.25	no
		Sitting	0.00	0.00	+ 0.00	no
	Pelvis alignment	Standing	-1.54	-0.67	+ 0.87	**yes**
		Sitting	-0.15	0.73	+ 0.88	no
	Quadriceps angle left		14.89	15.73	+ 0.84	no
	Quadriceps angle right		15.61	16.50	+ 0.89	**yes**
	Genu valgum / varum left		170.44	170.23	-0.21	no
	Genu valgum / varum right		171.27	170.85	-0.42	no

## Discussion

Numerous publications of the last decades discussed the anatomical and functional interactions of the temporomandibular system with global body posture [19, 32, 33]. Especially the role of occlusion is discussed controversially and dismissed by some authors [34]. However, due to the lack of high-quality studies, most recent systematic reviews concluded that there was insufficient scientific evidence, in order to support the thesis of interactions of TMD treatment with postural changes [19–21]. Exclusively cervical spine posture deviations seemed to correlate with symptoms of TMD as proposed by Chaves and coworkers in a single review [21], which is supported clinically especially in patients with hypermobile TMJs [35]. Prospective clinical trials investigating the effects of TMD therapy, especially by using occlusal appliances, are still lacking [19].

The present prospective clinical trial aimed to evaluate postural changes in patients with TMD after splint therapy using a non-invasive 3D-body scanner and a novel evaluation protocol. Repeated measurements showed good reliability, whereas the measured values were comparable to literature data [25]. Based on the statistical analyses of the present study only a limited effect of occlusal appliances on postural parameters was observed. However, significant changes before and after splint therapy were found for Forward head and Round shoulders angles (left/right) in both standing and upright sitting positions.

Angles regarding lower body posture (e.g., Knee flexion) were altered in the sitting position and were therefore not comparable between both positions (sitting / standing). Of these, only the Quadriceps angle of the right side was affected significantly after treatment and increased on average. An increase indicates a less superiorly directed pull of the quadriceps. However, this change was not found on both sides, it was lower than previously reported side differences of healthy individuals [25], and all average values (before and after treatment) were in the range of healthy individuals [36, 37]. Hence, this result does not appear to be clinically relevant. When comparing both positions, the changes in Frankfort plane, Lumbar lordosis, Thoracic kyphosis, Pelvic tilt, and alignment were not consistently significant and their directions were in opposite directions except for the Frankfort plane angle. Therefore, the authors of the present study assume that these changes are not of clinical relevance even though they were significant in either one position (sitting/standing) or on one side (left/right). In sitting position Frankfort plane median angles showed negative values with a statistically irrelevant decrease from T1 to T2, which was interpreted as a tendency to a posterior tilt of the head. As suggested by recent literature, backward inclination of the head and TMD symptoms are significantly associated in presence of cervical backward bending [38, 39]. However statistical analyses of the present study revealed a significant decrease of Frankfort plane angles in standing position, showing slightly positive values at T1, which approximated to zero at T2, whereas an angle of zero corresponded to a perfectly straight head position. Due to the definition of the Frankfort plane angle these results indicated a more upright position after treatment. Ignoring the tendency of decrease in the sitting position, alterations in the standing position could be considered as a treatment success. In contrast to the results of Frankfort plane angles, all changes in the Forward Head angle were consistent and statistically relevant both in standing and sitting positions. In general, Forward head angles increased (mean difference standing: +1.19°; sitting: +13.19°). A larger Forward head angle indicates that the head is less anteriorly drawn. Therefore, after treatment, the position of the head seemed to be more centered on the body’s core. In summary, these observations supported the finding of a less anterior-tilted head, which was also indicated by the changes of the Frankfort plane in the standing position. Further statistical analyses showed that patients with reduced mandibular mobility before treatment or with the RDC/TMD-diagnosis myofascial pain presented with the largest alteration of Forward head angle from T1 to T2. Even though there was no statistical significant relation between a specific RDC/TMD symptom and head posture [40], it is suggested that myogenous TMD pain can be responsible for muscle tone and postural adaptation in adjacent body segments [20] and therefore, the treatment of myofascial pain might have the biggest impact on global body posture. Furthermore, it has been shown that masticatory muscles are more pain tolerant in the presence of a more forward head posture [41, 42]. Accordingly, treating the cause of the pain may lead to a re-centering of the head posture. However, in recent literature, no relationship was found between the Forward Head angle and TMD pain, but it is suggested that neck pain and TMD pain might be associated [42]. Further research on compensatory mechanisms and the influence of TMD pain on body posture is needed.

In contrast to Forward head angles, the Round shoulder angles decreased (mean difference standing, right: -4.74°; standing, left: ‐4.30°; sitting, right: ‐11.14°; sitting, left: ‐5.56°). A smaller Round shoulders angle indicated more anteriorly drawn shoulders. A more forward shoulder position may lead to muscle imbalance by the shortening of the anterior shoulder muscles [43] and thereby, an alteration of scapular and glenohumeral orientation may lead to biomechanical changes [44, 45]. Subsequently, this might increase the risk of musculoskeletal pain or functional impairment, such as limited range of motion [46]. Some authors suggested that this misalignment of the shoulders with the body’s core is commonly induced by repetitive overhead activities, backpack carriage, bad habits, computer and laptop use, and prolonged study hours, among other factors [43, 46–48]. Therefore, the results of the present study could be distorted by the aforementioned confounding factors. A limitation of the present study might be the influence originating from unknown factors, particularly found in the homogenous collective of mainly female TMD patients. However, it has already been described that TMD symptoms are more prevalent in females [49], but conclusions on the basis of the present results might only been drawn with regard to functionally impaired individuals and not healthy subjects. Within the present study design, all full body scans were performed without the application of occlusal splints, since various studies described the instantaneous adaptation of body posture and the reversal back to habitual posture after removal of the occlusal appliance [50, 51]. As a result, it is solely possible to conclude that splint therapy itself had very limited effect on permanent body posture. Future studies should repeat the proposed study setup with comparative analyses on additional scan groups wearing the occlusal appliance. Besides static changes, recent literature suggests enhanced mobility and athletic performance through splint therapy [50, 52, 53]. Utilizing the scanning procedure of this study an evaluation protocol for dynamic parameters, like the range of full-body motions, could be established to evaluate the influence of dental splints on mobility in a standardized manner.

Another limitation of this study might be the classification of TMD diagnoses according to the RDC/TMD criteria rather than the newer DC/TMD protocol. However, for a more robust statistical analysis, the diagnoses and symptoms were grouped (presence or absence of restricted jaw mobility, joint noises, myofascial pain, and combined pain), as already proposed by other authors [27]. Presumably, though, application of the DC/TMD protocol would have resulted in the same group compositions. Nevertheless, follow-up studies should examine patients according to DC/TMD criteria. Although the female predominance across all groups corresponds to the general TMD gender distribution, the average age of participants was on the lower end of the typical TMD demographic. Many older patients either did not consent to the study, had preexisting orthopedic conditions or had already received splint treatment, which prevented enrollment. Furthermore, the enrollment of a relatively small sample size may have hindered the detection of very small effects on global posture. However, since most changes were not consistent in direction when comparing standing and sitting positions, the authors believe that the impact on more caudal body segments is unlikely.

When evaluating body posture, intra-individual variability may present a confounding factor. To reduce its influence, we chose an analytical method that had already demonstrated high repeatability when comparing scans of healthy subjects taken 24 h apart [25]. Additionally, scans were performed in triplicate to minimize random effects. By repeating the measurements, we were able to average the values to obtain reliable data. Furthermore, the analyses were performed in duplicate by one blinded observer. The authors of this study believe that this protocol should lead to a significant reduction of this non-avoidable confounding factor.

The present study also lacks a healthy control group. Consequently, it is possible that the measured changes in body posture after splint treatment could also occur in healthy subjects wearing a splint. To the authors’ knowledge, the reorganizational effects on neuromuscular-functional patterns of occlusal splints in healthy subjects remain unclear. Due to the lack of this information, a control group wearing a splint could have introduced a confounding factor. Furthermore, it is questionable whether healthy subjects would adhere to the required wear time due to the lack of symptoms. To avoid both potential confounding factors, the authors of this study decided against including a healthy control group and instead analyzed two groups of symptomatic patients comparatively.

In summary, the results of the present study showed that the influence of splint therapy on static body posture is limited. Within the present study only changes in cervicocranial parameters were found, which was comparable to the results of recent reviews. These changes indicated a straighter head position, but more anteriorly tilted shoulders. The latter might be influenced by the functional impairment of the homogenous subpopulation of mainly female TMD subjects. Future trials should investigate posture changes of healthy and impaired volunteers before and after the removal of the occlusal appliance with consideration of dynamic parameters.

## Conclusion

The present prospective, clinical trial aimed to evaluate the influence of occlusal splints on static, global body posture using an established, non-invasive, and reliable study protocol. The findings of this study show that the influence of occlusal splints on global posture is limited and only a small effect on cervicocranial parameters was found. After a regular treatment period, the average head position seemed to be more centered on the body’s core, while the shoulders were tilted more anteriorly. Future studies should investigate the influence of splint therapy by using dynamic scans in order to better determine dynamic functional changes while wearing the occlusal appliance and without it.

## Data Availability

Data sets generated during the current study are available from the corresponding author on reasonable request.
